# Fusion plasmid enhanced the endemic extensively drug resistant *Klebsiella pneumoniae* clone ST147 harbored *bla*_OXA-48_ to acquire the hypervirulence and cause fatal infection

**DOI:** 10.1186/s12941-022-00551-1

**Published:** 2023-02-14

**Authors:** Chao Liu, Pengcheng Du, Ping Yang, Ming Lu, Ning Shen

**Affiliations:** 1 Department of Infectious Disease, Peking University Third Hospital, Beijing, China; 2 Center of Infectious Disease, Peking University Third Hospital, Beijing, China; 3 Qitan Technology Ltd., Chengdu, China; 4 Department of Pulmonary and Critical Care Medicine, Peking University Third Hospital, Beijing, China; 5 Institute of Medical Technology, Peking University Health Science Center, Beijing, China

**Keywords:** Hypervirulent *Klebsiella pneumoniae*, Extensively drug resistant, ST147, Fusion plasmid, *Bla*_OXA-48_

## Abstract

**Background:**

*Klebsiella Pneumoniae* (Kp) sequence type (ST) 147 has emerged globally and spread rapidly, particularly the extensively drug resistant (XDR) isolates. However, the infections caused by this subtype is rare reported in China for now. The clinical, microbiological and genomic characteristics are unclear.

**Methods:**

A systemic retrospective study was conducted in a Chinese tertiary hospital. Clinical information of the infection cases was collected, and whole-genome sequencing and phenotypic experiments were performed on the ST147 isolates. The resistance and virulence genes were identified, and the plasmids harboring these genes were further studied.

**Results:**

Six ST147 isolates from six patients among 720 available clincial Kp isolates were detected. Notably, two isolates, PEKP4035 and PEKP4265, represented both XDR and hypervirulence by acquiring *bla*_OXA-48_, *bla*_CTX-M-15_ and key virulence genes, *iucA* + *rmpA2*, representing no fitness cost and resulting fatal infection. Four of the six ST147 isolates presented with more nucleotide differences, whereas the PEKP4035 and PEKP4265 both isolated from the intensive care unit possessed 20 single nucleotide polymorphisms among one year, indicating the prolonged survive and transmission. Interestingly, the two isolates harbored the same fused plasmid composed of *sul2* and *iucA* + *rmpA2*, which might be generated by recombination of a plasmid like KpvST101_OXA-48 with the pLVPK plasmid via IS*26*. Besides, two ~ 70 kb plasmids conferring multiple-drug resistance were also identified among the two isolates, which presented resistance genes including *bla*_OXA-48_, *bla*_CTX-M-16_, *strA* and *strB*. Interestingly, we reported that *bla*_CTX-M-15_, a common resistance gene within ST147, has successfully transferred into the chromosome by IS*Ecp1*.

**Conclusions:**

XDR hypervirulent ST147 Kp is emerging, suggesting enhanced surveillance is essential.

**Supplementary Information:**

The online version contains supplementary material available at 10.1186/s12941-022-00551-1.

## Introduction

*Klebsiella pneumoniae* (Kp) is an increasingly important pathogen for the capture of causing various infections [1]. In addition to the disturbing resistances to multiple last-line antimicrobial agents, it is also emerging as a key trafficker of various pathogenicity genes due to its high degree of genome plasticity [2]. Kp has successfully evolved into two pathotypes termed the hypervirulent *K. pneumoniae* (hvKp) and classical *K. pneumoniae* (cKp) [3].

Both of the two pathotypes presented with distinct characteristics. Previously, the hvKp commonly isolated from the community-acquired liver abscess and was sensitive to most of the antibiotics [4]. From the genetic trait of the hvKp, it is clear that pLVPK/pVir-CR-HVKP4 virulence plasmid was the common contributor [3, 5, 6]. These virulence plasmids were mainly harbored by sequence type (ST) 23 hvKp previously, however, ST11 hvKp harboring similar virulence plasmid has emerged and spread worldwide in recent years. In contrast, cKp featured by high antimicrobial resistance has been prevalent in clinical settings for decades, and was associated with multiple subtypes including ST11, ST258, ST15, etc. The prevalent population of cKp formed a large gene pool for the intra- and inter-species gene exchange and might accelerate the emergence of subtypes with new genetic and phenotypic characteristics.

As the representative cKp, the globe high-risk clone ST147 that initially possessed the mutations within quinolone-resistance determining regions (QRDRs) including S83I in *GyrA* and S80I in *ParC*, and *bla*_CTX-M-15_ conferring resistance to β-lactams [7]. Recently, ST147 Kp is also notorious for the acquisition of the *bla*_VIM_ [8] and *bla*_KPC_ [9] and *bla*_NDM_ [10–12], which is posing great threat to the public health [13]. This subtype is prevalent in the India and North Africa and spread to the worldwide [13], whereas few study reported infections by ST147 in China. Importantly, previous studies demonstrated that hvKp defined as the combination of the *iucA, iroB, rmpA, rmpA2 or peg344* is replacing the cKp as the dominant pathotype in the nosocomial and healthcare-associated infection [6] and the cKp is increasingly acquiring the virulence-associated genes in China [6, 14]. Recent study reported that MDR-ST147-hvKp is spread in patients with severe COVID-19 in Italy [15]. However, there is few data focused on the clinical and genomic characteristics of multidrug-resistance (MDR) and hypervirulent ST147 clone in China.

Here, we performed a four-year genome surveillance of Kp in a hospital in China, and discovered novel ST147 Kp characterized a fusion plasmid that encodes both antimicrobial resistance and hypervirulence. Within this ST147 clone, we also observed coharboring of the *bla*_CTX-M-15_ and *bla*_OXA-48_ conferring extensively drug resistance (XDR), and resulting in fatal infection. Additionally, we found diverse genetic context of the *bla*_CTX-M-15_ within ST147 and reported that *bla*_CTX-M-15_ was integrated into the chromosome from plasmid via recombination mediated by the IS*Ecp1*.

## Materials and methods

### Enrolled bacterial isolates and clinical characteristics

From the genomic surveillance conducted within the Peking University Third Hospital, seven hundred twenty *Klebsiella. spp* were enrolled from 2017 to 2021, among which 6 isolates were examined. All strains were stored at − 80 °C. Strains were initially identified by the MALDI-TOF mass spectrometry and then confirmed by Vitek compact 2 system. The clinical characteristics were obtained from electronic medical records. Basic demographic characteristics, underlying diseases, antimicrobial agent exposure within 90 days, infection type, use of invasive devices and outcomes were collected. Additionally, the Charlson comorbidity index (CCI) was calculated. The protocol for this study was approved by the Peking University Third Hospital Ethics Committee (M2021238), and the Guidelines for Human Experimentation (PRC) were followed throughout. Community-acquired infection (CAI), healthcare-associated (HCAI) and nosocomial infection (HAI) were identified as previously described [6]. Multidrug resistance (MDR) and extensive drug resistance (XDR) were defined as previously described [16].

### Antimicrobial susceptibility testing (AST)

AST was performed on the 6 isolates by the Vitek 2 or disk diffusion test. The antimicrobials tested were cefepime (FEP), ceftazidime (CAZ), aztreonam (ATM), imipenem (IPM), meropenem (MEM), piperacillin/tazobactam (TZP), cefperazone-sulbactam (CSL), amikacin (AMK), tobramycin (TOB), colistin (COL), levofloxacin (LVX), ciprofloxacin (CIP), minocycline (MNO), tigecycline (TGC), polymyxin (POL), cefiderocol (FDC), ceftazidime/avibactam (CZA) and trimethoprim/sulfamethoxazole (SXT). AST results were interpreted using 2020 Clinical and Laboratory Standards Institute (CLSI) guideline breakpoints and the tigecycline was followed by the FDA recommendation.

### Virulence-associated phenotype and growth curve

HvKp were defined by the presence of some combination of *peg-344, iroB, iucA, rmpA,* or *rmpA2* [17]. Hypermucoviscous phenotype was defined by a positive string test as described previously [18].

### Serum killing assays

Serum killing assays of all ST147 strains were conducted as previously described [19]. Briefly, we collected bacterial suspensions and mixed them with healthy human serum at 1:3. The mixture was agitated at 37 °C and the number of the clone was recorded at different time (0 h, 1 h, 2 h, 3 h, respectively). Survival percentages at each different time points were calculated three times and the results were interpreted by previous definition [20].

### Biofilm formation capacity

Biofilm formation capacity was evaluated in 96 well plate as previously described [21]. Briefly, bacterial suspensions were inoculated in a 96-well plate followed by 48 h of incubation at 37 °C. Strains were washed with distilled water three times and dyed. Subsequently, the plate wells were incubated with 200 μl of 1% Crystal Violet dye for 20 min and further washed twice. We added 200 μl of 95% ethanol into each well, and the optical density (OD) at 570 nm of the tested isolates was measured and recorded as A. The wells added into LB medium were used as the negative control (Ac). The results were interpreted by the following criteria: (1) No biofilm producer (−), A ≤ Ac; (2) weakly positive (+ +), Ac < A ≤ 2Ac; (3) Moderately positive (+ +), 2Ac < A ≤ 4Ac; and (4) Strongly positive (+ + +), A > 4Ac.

### Growth curve

To compare the fitness cost, growth curve was conducted. Briefly, the ST147 strains carrying fusion plasmid cultured overnight and then were diluted to an optical density (OD) 600 of 0.1 and grown at 37 °C in triplicate for 16 h. The OD600 of the cell density was determined every per hour and the growth curve was conducted by the GraphPad Prism version 7.

### Galleria mellonella model

The virulence phenotype was evaluated using Galleria mellonella (insects of approximately 300 mg). Strains were adjusted using physiological saline to 1 × 10^6^ CFU/mL and then injected into the Galleria mellonella, as described previously [19]. The survival rate was recorded at 12, 24, 36, 48, 60 and 72 h, respectively and all strains were tested three times.

### Plasmid transferability and stability

To clearly understand the plasmid stability, we subcultured strains serially to the 10th passage [22]. The descendants were evaluated by the AST.

For identifying the transferability of the fused plasmid, *E. coli J53* was selected as the recipient to perform the conjugation experiments [23]. Briefly, the donor and recipient strain mixed at a ratio of 1:1 and then co-cultured in LB broth overnight. Subsequently, the mixture was spotted on the MacConkey agar containing 320 mg/liter sulfanilamide and 200 mg/liter of sodium azide. After overnight culture, the transconjugants were finally screened. To further understand the transferability of the *bla*_OXA-48_ plasmid, we also used the *E. coli J53* as the recipient and the meropenem (2 mg/liter) was chosen as the screening antibiotic.

### WGS and bioinformatics analysis

The whole genomic of all the ST147 strains was extracted and sequenced by the NovaSeq 6000 platform (paired-end library, 150-bp). The low-quality reads were removed by fastp (https://github.com/OpenGene/fastp). The left clean data were de novo assembled by SPAdes v3.13 and annotated using the Prokka software [24]. resistance genes, virulence genes, insertion sequences (ISs) and plasmid replicon types were identified by ResFinder [25], Virulence Factor Database [26], IsFinder [27], and plasmidFinder [28] database.

To further understand the genomic characteristics of the MDR-hvKp strains, the PEKP4035 and PEKP4265 strains were sequenced by the long-read sequencing platform-MinION to acquire the complete genomes via de novo assembly with a hybrid strategy according to published methods, and assembled using Unicycler [29]. Additionally, to get the genetic context of the *bla*_CTX-M-like_ within the ST147 strains, we also sequenced the P4 and PEKP4243 to get the complete genomes. *Kleborate* software was used to determine STs and to detect *Yersiniabactin, Colibactin, Aerobactin, Salmochelin*, of K and O locus serotypes, associated ICEKp, and the number of AMR classes [30].

The published genome data of the ST147 *K. pneumoniae* in the GenBank database (accessed on September 1, 2021) were enrolled. Sequencing reads were mapped to the *K. pneumoniae* HS11286 (accession no.: NC_016845) using bowtie 2 [31] v2.2.8 and the single nucleotide polymorphisms (SNPs) were identified by using Samtools v1.9 and combined according to the reference genome (*K. pneumoniae* HS11286) using the previously constructed iSNV-calling pipeline (https://github.com/generality/iSNV-calling). High-quality SNPs (> 5 reads of mapping quality  > 20) were retained and the recombination sites were further identified by Gubbins [32]. The concatenated sequences of filtered polymorphic sites conserved in all genomes (core genome SNPs, cgSNPs) were used to perform phylogenetic analysis using maximum likelihood method by FastTree software [33].

### Nucleotide sequence accession numbers

All the six ST147 genome sequences have been deposited in the NCBI GenBank database under the accession number PRJNA838419.

## Results

### Emergence of the MDR-ST147-hvKp causing fatal infection

We obtained six ST147 Kp isolates from six patients among 720 available Kp isolates from 454 patients in total. The age of the patient ranged from 53 to 81y. Four cases were defined as HAI and two were HCAI. Five of the six cases (83.3%) were associated with CCI ≥ 3 and two cases (33.3%) suffered from sepsis and shock (Table 1). Four of the six isolates presented MDR phenotypes and two were XDR presenting resistance to quinolones, aminoglycosides, carbapenems and β-lactamase/inhibitors (Table 1 and Additional file 1: Table S1). All the six isolates showed no hypermucoviscosity (Table 2). The biofilm formation capacity presented within all the isolates and no significant fitness cost was shown among all the isolates (Additional file 1: Figure S1, Table 2). Four of the six isolates belonged to KL64 serotype and all of the six isolates displayed sensitivity to the serum (Table 2 and Additional file 1: Figure S2). Interestingly, four ST147 isolates (66.7%) represented resistance to tigecycline, greatly challenging the clinical practice (Additional file 1: Table S1).

**Table 1:** Clinical characteristics of the ST147 Kp strains

Patient	P4	PEKP4035	PEKP4225	PEKP4243	PEKP4265	PEKP5078
Age	64	97	53	66	81	81
Sex	male	male	female	male	male	female
Specimen	urine	secretion	urine	abdominal fluid	blood	sputum
Isolation date	2017/11/10	2018/10/11	2017/1/4	2019/7/5	2019/8/16	2021/4/20
Department	Urology Surgery	ICU	Urology Surgery	General Surgery	ICU	Neurology
Underlying diseases	Urothelial carcinoma	Cerebrovascular disease, urinary disease, hypertension, fracture;	Diabetes mellitus; endometrial cancer;	Diabetes mellitus; duodenal ulcer; gastrectomy;	Pancreatic cancer; diabetes mellitus; hypertension; iron deficiency anemia;	Hypertension; coronary heart disease; diabetes mellitus; dementia; cerebrovascular disease; depressive states;
Antibiotic agent exposure	Levofloxacin; Ertapenem;	Isopamicin; cefepime; Minocycline; Meropenem; Cefoperazone/Sulbactam; Imipenem; Linezolid; Tigecycline; Vancomycin;	Levofloxacin; Ciprofloxacin; Ertapenem;	Cefdinir; Piperacillin/Sulbactam	Cefoperazone/Sulbactam; Imipenem;	Piperacillin/Sulbactam;
Incubation	Urinary catheter	Gastrostomy tube; urinary catheter; PICC	Urinary catheter	Urinary catheter; Gastrostomy tube	Gastrostomy tube; PICC;	Gastrostomy tube; Urinary catheter;
Infection type	HCAI	HAI	HAI	HCAI	HAI	HAI
Metastatic infection	No	Yes	No	No	Yes	No
Mechanical ventilation	No	Yes	No	No	No	No
Vasoactive drugs	No	Yes	No	No	Yes	No
CCI ≥ 3	No	Yes	Yo	Yes	Yes	Yes
SOFA > 6	No	Yes	No	No	Yes	No
Septic Shock	No	Yes	No	No	Yes	No
Outcome in 30 days	Survived	Death within 7 days	Survived	Survived	Death within 2 days	Survived

*PICC* Peripherally Inserted Central Catheter, *HAI* Hospital-acquired Infection, *HCAI* Healthcare-associated Infection

**Table 2:** Microbiological and genomic characteristics of the ST147 strains

Characteristics	P4	PEKP4035	PEKP4225	PEKP4243	PEKP4265	PEKP5078
Hypermucoviscosity	Neg	Neg	Neg	Neg	Neg	Neg
MDR phenotype	Pos	Pos	Pos	Pos	Pos	Pos
CR phenotype	Neg	Pos	Neg	Neg	Pos	Neg
Biofilm	+	+	+	+ +	+	+
Genotype	KL64- O2v1	KL64- O2v1	KLn*- O2v1	KL64- O2v1	KL64- O2v1	KL106- O2v1
Resistance marker	*bla*_*CTX-M-55*_	*bla*_OXA-48_; *bla*_CTX-M-16*;*_	*bla*_*CTX-M-55*_	*bla*_*CTX-M-3*_	*bla*_OXA-48_; *bla*_CTX-M-15*;*_* bla*_CTX-M-16_	*bla*_*CTX-M-3*_
Virulence replication	–	IncHI1B-IncFIB	–	–	IncHI1B-IncFIB	–
Capsule regulator	–	*rmpA2*	–	–	*rmpA2*	–
Yersiniabactin-ST	–	YbST20-2LV (ybt10- ICEKp4)	–	–	YbST20-2LV (ybt10- ICEKp4)	–
Aerobactin-ST	–	AbST63 *iucA1B15C1D1iutA1*	–	–	AbST63-1LV *iucA1B15C1D1*^*#*^*iutA1*	–

^*^:KLn: unknow serotype

Pos: Positive

Neg: Negative

Importantly, the two XDR serotype KL64 isolates, PEKP4035 and PEKP4265, also showed hypervirulence in the Galleria mellonella model but without hypermucoviscous phenotype. The two patients infected by the two isolates were critically ill (SOFA > 6) and died within 7 days after admission (Table 1, Additional file 1: Figure S3). Therefore, the two isolates displayed both carbapenem-resistance and hypervirulence phenotype, and were defined as CR-hvKp.

### Phylogenetic analysis, differences of virulence gene and resistance gene profiles

To assess the phylogenetic relationship of our ST147 isolates, we collected 473 published genome sequences of ST147 Kp isolates in addition to the 6 we sequenced. A maximum likelihood phylogenetic tree was constructed using all the 479 genomes based on 16,994 hqSNPs. Our six ST147 isolates were clustered in three distinct branches (Additional file 1: Figure S4). The two XDR hvKp isolates were clustered together with other five published ST147 hvKp genomes. The isolation interval of PEKP4035 and PEKP4265 was almost one year, and their genomes differed by 20 hqSNPs, suggesting the XDR-hvKp might be persistently existed in the hospital (Additional file 1: Table S2). Most of the 479 ST147 genomes (462, 96.2%) carried resistance genes against three or more classes of antimicrobial agents and were denoted as MDR. Forty-six (9.6%) carried at least one of the five virulence associated genes including *peg-344, iroB, iucA, rmpA,* and *rmpA2*, and were denoted as hvKp, all of which were also MDR. In addition, these hvKp were clustered with cKp in different branches, indicating multiple horizontal transfer events of virulence genes and acquisition among MDR ST147 cKp, by which the MDR-ST147-hvKp evolved (Additional file 1: Figure S4).

**Fig. 1:**
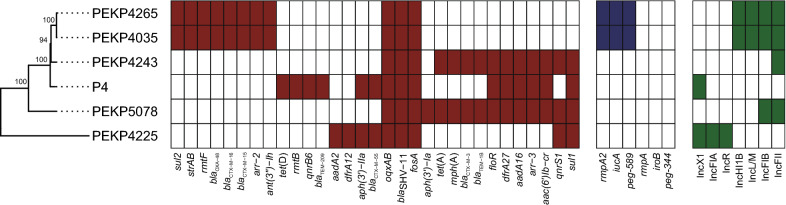
Phylogenetic analysis of ST147 Kp in our hospital correlate with the distribution of resistance genes (red-white heatmap), virulence genes (blue-white heatmap) and plasmid types (green-white heatmap)

The virulence genes including *iucABCDiutA*, *peg-589* and *rmpA2* were identified in PEKP4035 and PEKP4265, which were in line with the hypervirulence phenotype. In contrast, none virulence gene was discovered in other four cKp. The two CR-hvKp isolates carried the same resistance genes but the four cKp isolates represented different resistance gene profiles. The resistance genes including *bla*_OXA-48_, *bla*_CTX-M-15_, *bla*_CTX-M-16_, *sul2*, *arr*-2, *strA-strB*, *ant(3'')*–*Ih* and *rmtF* were identified in the two CR-hvKp and in line with their XDR phenotype, and none of these resistance genes were identified in the four ST147 cKp. Only three resistance genes were shared by the two CR-hvKp isolates and four cKp isolates, including *oqxA*-*oqxB*, *bla*_SHV-11_ and *fosA*. These results indicated different phylogenetic features, resistance gene and virulence gene profiles of the two ST147 CR-hvKp compared with ST147 cKp in our hospital (Fig. 1).

### Fusion plasmid encoding both virulence genes and resistance genes

**Fig. 2:**
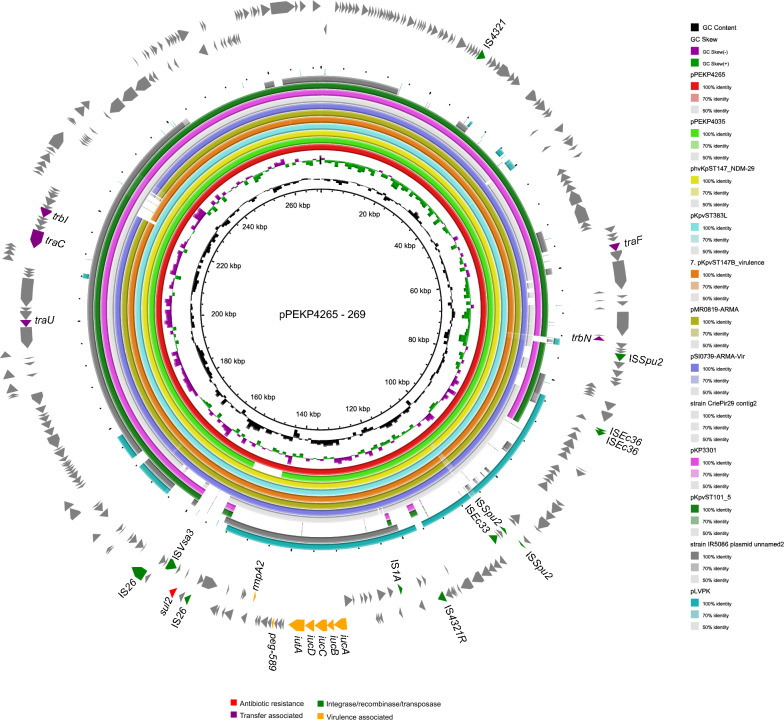
Comparison between fused plasmid pPEKP4265-269 encoding *sul2 and iucA* + *rmpA2* found in this study and similar plasmids found in the online NCBI database. The outmost circle of arrows indicated the genes of reference plasmid pPEKP4265-269 used for comparison (red: AMR genes; green: integrase, recombinase, and transposase genes; purple: transfer associated genes; orange: gray: other functions)

**Fig. 3:**
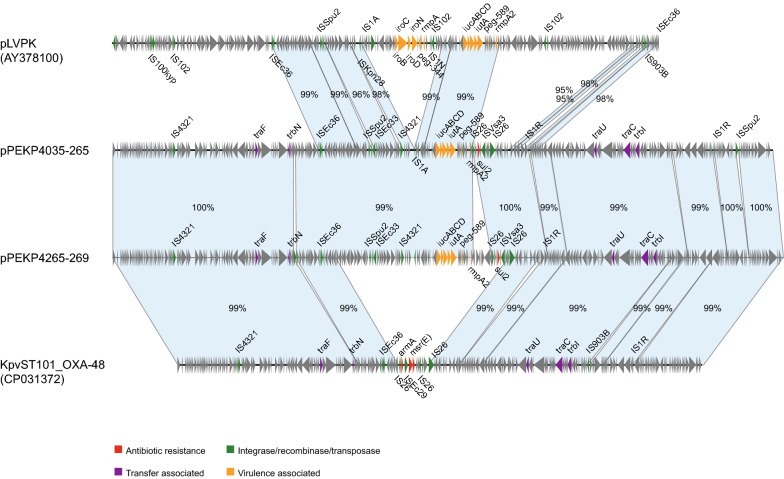
Linear plasmid sequence comparison of plasmid pLVPK, pPEKP4035-265, pPEKP4265-269 and KpvST101_OXA-48. The matched regions between two sequences are displayed by light blue blocks. The arrows represent the genes related to resistance and transfer (red: AMR genes; green: integrase, recombinase, and transposase genes; purple: transfer associated genes; orange: virulence associated genes; gray: other functions)

We then obtained the complete sequences of the two XDR-ST147-hvKp isolates, PEKP4035 and PEKP4265. Two plasmids, pPEKP4035-265 and pPEKP4265-269, which were highly similar with each other only differed by small indels (Fig. 2), were found to encode the virulence genes, including *iucABCDiutA*, *peg-589* and *rmpA2*. The plasmid pPEKP4035-265 was 265-kb in size and pPEKP4265-269 was 269-kb in size, and both comprised of the IncFIB and IncHI1B types of plasmid replicons. The large difference was a 7.5-kb insertion identified in pPEKP4265-269 encoding hypothetical proteins. The AMR gene *sul2* was also identified in the two plasmids. These plasmids were highly similar with plasmid KpvST101_OXA-48 (accession number: CP031372, 93.7% coverage with 98.3% identity) (Fig. 3). The ~ 70-kb fragment on pPEKP4265-269 encoding the virulence genes was found to replace the ~ 8-kb resistance regions with the genetic context of IS*26*-*armA*-IS*Ec29*-*msr*(E)-IS*26* on plasmid KpvST101_OXA-48. This fragment was highly similar with the corresponding regions of virulence plasmid pLVPK (96% ~ 99% identity), and an IS*26* element was also found in the right side of this fragment (Fig. 3). Therefore, this plasmid might be generated by recombination of a plasmid like KpvST101_OXA-48 with the classic virulence plasmid of hvKp via IS*26*.

**Fig. 4:**
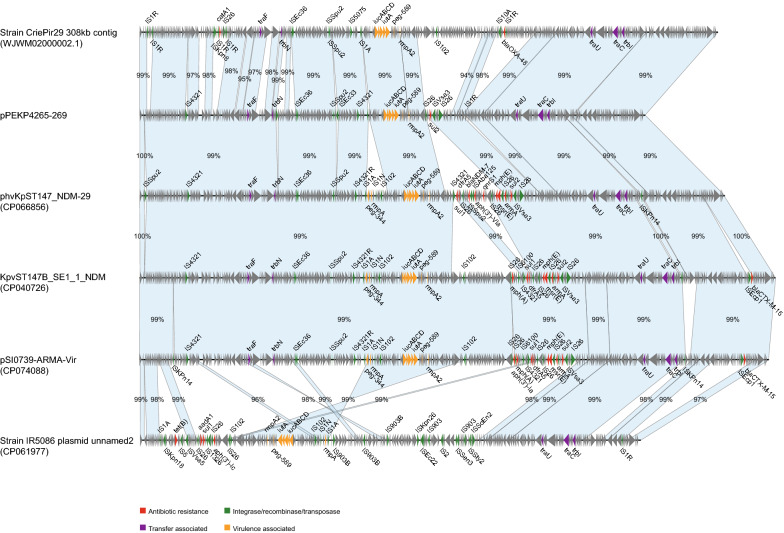
Linear plasmid sequence comparison of plasmids similar with pPEKP4265-269. The matched regions between two sequences are displayed by light blue blocks. The arrows represent the genes related to resistance and transfer (red: AMR genes; green: integrase, recombinase, and transposase genes; purple: transfer associated genes; orange: virulence associated genes; gray: other functions)

The similar fusion plasmids were also identified among published data in NCBI, like phvKpST147_NDM-29, KpvST147B_SE1_1_NDM, pSI0739-ARMA-Vir, an unnamed plasmid of strain IR5086, and a 308-kb contig of strain CriePir29 (Fig. 4). These plasmids encoded a number of resistance genes, particularly in phvKpST147_NDM-29 the *bla*_NDM-7_ gene was identified. The resistance regions on these plasmids comprised of resistance genes and multiple types of ISs including IS*26*, which were well known as common recombination sites for resistance elements. Therefore, this type of fusion plasmid encoded both virulence genes and resistance islands, and had the potential ability to acquire more resistance genes.

### Co-harboring of virulence plasmid and MDR plasmid

In addition to the virulence plasmids, a ~ 70-kb MDR plasmid was also identified among the two ST147 MDR hvKp, named pPEKP4035-70 and pPEKP4265-70. This plasmid comprised of the IncL/M type of plasmid replicon, and resistance genes including *bla*_OXA-48_, *bla*_CTX-M-16_, *strA* and *strB* (Additional file 1: Figure S5). This plasmid accounted for the XDR phenotype including resistances to carbapenems and β-lactams. The *bla*_OXA-48_ was located within the resistance element comprised of IS*10A*, IS*1R*, *bla*_OXA-48_ and IS*10A*, and the other three genes were located within the resistance element comprised of IS*Ecp1*, IS*26*, *bla*_CTX-M-16_, *strA* and *strB*. The fusion plasmid encoding virulence genes and resistance gene *sul2*, and the IncL/M type plasmid encoding resistance gene *bla*_OXA-48_ did not transfer into the recipient in the conjugation assays, and could persistently exist during continuous passages.

### Diverse genetic context of the *bla*_CTX-M-15_ within the ST147 isolates

**Fig. 5:**
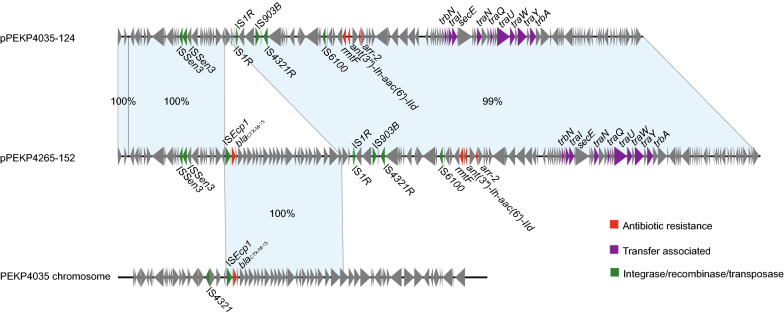
Linear plasmid sequence comparison of plasmid pPEKP4035-124, pPEKP4265-152 and corresponding regions on the chromosome of PEKP4035. The matched regions between two sequences are displayed by light blue blocks. The arrows represent the genes related to resistance and transfer (red: AMR genes; green: integrase, recombinase, and transposase genes; purple: transfer associated genes; orange: gray: other functions)

The PEKP4035 and PEKP4265 both carried *bla*_CTX-M-15_. Previous study reported that *bla*_CTX-M-15_ located on IncF or IncR plasmids [13]. A 152-kb IncFII type plasmid, pPEKP4265-152, were discovered in the isolate PEKP4265 and harbored *bla*_CTX-M-15_ adjacent to an IS*Ecp1* element (Additional file 1: Figure S6). Three other resistance genes, *arr-2*, *aac(6')-IId* and *rmtF* were also identified in a resistance region 50-kb away from *bla*_CTX-M-15_. The similar IncFII plasmid, pPEKP4035-124, was also found in the other XDR hvKp isolate PEKP4035. Interestingly, *bla*_CTX-M-15_ was absent in plasmid pPEKP4035-124, and was identified in the chromosome of PEKP4035. By the comparative analysis, we found that a 27.8-kb fragment encoding *bla*_CTX-M-15_ in pPEKP4265-152 was lost in pPEKP4035-124 and inserted into the chromosome of PEKP4035. These results indicated the recombination of this fragment between chromosome and plasmid caused the relocation of *bla*_CTX-M-15_, which might be mediated by the IS*Ecp1* (Fig. 5).

## Discussion

To the best of our knowledge, this is the first report for theemergence, clinical and genomic characteristics of the MDR-ST147-hvKp in a large hospital in China. Although the ST147 accounts for less than one percent during four years in the study in the hospital, more than half of the MDR-ST147 isolates presented with tigecycline non-susceptibility and one third MDR-ST147 isolates harbored key virulence genes *iucA* and *rmpA2*. What makes things worse is that XDR-ST147-hvKp emerged and resulted fatal infection. More importantly, it is noted that the fusion plasmid co-encoded the resistance and hypervirulence phenotype is emerging, alarming that it might enhance the MDR or XDR ST147 clone to confer with the hypervirulence and then transmit within the hospital environment, posing great threat to the public health. Additionally, we reported that the genomic context of the *bla*_CTX-M-15_ is diverse and firstly found that *bla*_CTX-M-15_ located in the chromosome within the ST147 isolates, forming stable MDR phenotypes. These findings are concerning and have significant implications for patient care and outcomes.

ST147 is notorious as the high risk cKp with high resistance globally, which is special endemic in India, Italy, Greece, and North African countries [13]. Previous studies demonstrated that ST147 harboring various resistance genes triggered various outbreaks [34, 35]. However, few studies focused on the ST147 in China. Wang, et al. [36] firstly reported an outbreak of a nosocomial NDM-1-Producing ST147 Kp in China and all the patients presented with no history of traveling to endemic regions or receiving any healthcare abroad. Additional studies from our country also unclarified the recent traveling history of the enrolled patients who infected with ST147 [37], suggesting that the origin and transmission is not clear. It seems that ST147 isolated from the Europe tended to acquire *bla*_NDM-like_ [10, 11, 34]. However, *bla*_OXA-48_, not the *bla*_NDM-like_, regarded as the predominant carbapenemase genes within the ST147 isolates in our hospital.

It is noted that the clinical characteristics of the ST147 clone also needed for further study [13]. Previous studies reported that ST147 mostly isolated from the rectal swabs, which is highly associated with colonization, not infection [10, 11]. The detection rate of the ST147 in our hospital is lower than the classical predominant cKp, ST11 [38]. The patients were middle-aged and suffered from various underlying diseases, and all the isolates presented resistances to all first-line agents, similar to the previous studies [11]. Importantly, the emergence of the XDR-ST147-hvKp presented with tigecycline non-susceptibility triggered fatal infection in the hospital, greatly challenging the clinical practice.

In the study, the two XDR-hvKp presented with KL64 and resulted fatal infections within 7 days, which was in line with the hypervirulence in vivo model. Interestingly, the two isolates did not possess hypermucoviscosity phenotype, similar to previous study [11], suggesting that regulation of the hypermucoviscosity phenotype formation needs further studies. Previous study demonstrated that thickness of the capsule might be serve as physical barrier for acquiring external genes [39]. ST147 has divided into two main clades with distinct KL subtypes [40]. The predominant is KL64, firstly emerged in 1994 and rapidly disseminated worldwide, and highly associated with various carbapenemases [40]. The other is KL10, predominantly found in Asian countries, closely related with *bla*_NDM-like_ and *bla*_OXA-48_ [40]. Interestingly, two XDR ST147 isolates we obtained presented with KL64 acquired *bla*_OXA-48_ and key virulence genes (*iucA* + *rmpA2*), and conferred with hypervirulence and carbapenem resistant phenotype.

Fusion plasmid plays great roles for the convergence of the resistance and hypervirulence within either cKp or hvKp strains [41, 42]. In the study, fusion plasmids of the PEKP4035 and PEKP4265 might be generated by multi-recombination events of a plasmid like KpvST101_OXA-48 with the pLVPK via IS*26*, not the pVir-like plasmid. Previous study reported that pLVPK-like plasmid acquired *bla*_KPC-2_ within ST23 Kp mediated by the IS*26* element [43]. Our previous study also demonstrated that IS*26* contributed to the formation of the MDR-hvKp [44]. Interestingly, the two XDR-hvKp strains co-harbored fusion plasmids that possessed the backbone of the KpvST101_OXA-48 plasmid and IncL/M plasmid that presented with *bla*_OXA-48_, suggesting that continuous dynamic monitoring of the fusion plasmids by WGS is essential. During about one year, PEKP4035 and PEKP4265 were detected in the ICU department, and different by 20 SNPs. One possible reason is that the XDR and hypervirulence triggered the prolonged transmission. The other possibility is that multiple plasmids harbored *bla*_OXA-48_, *bla*_CTX-M-like_, *rmtF* and *iucA* + *rmpA2* could co-transfer into other Kp strains. These hypothesis need large-scale systematic clinical and genomic surveillance to further understand the transmission route.

The global dissemination of ST147 occurred primarily driven by QRDR mutations and the acquisition of *bla*_CTX-M-15_ located on IncF or IncR plasmids [13, 45]. The location of *bla*_CTX-M-15_ in the chromosome has already been reported in *K. pneumoniae* and other Enterobacterales [46]. In the study, we found that the two isolates PEKP4265 and PEKP4035 possessed *bla*_CTX-M-15_. Interestingly, the genetic contexts of the *bla*_CTX-M-15_ in the two isolates are different. One is located in the IncFII plasmid and the other is in the chromosome. These results indicated that *bla*_CTX-M-15_ might be successfully harbored by both of the plasmid and chromosome in ST147 *K. pneumoniae* and contribute to the dissemination of ST147.

## Conclusions

In summary, XDR-ST147-hvKp is emerging and caused fatal infection. Whole genomic sequencing enhanced the surveillance of genetic context of the resistance genes and the hybrid plasmids converged the resistance and virulence.

## Supplementary Information

**Additional file 1: Table S1.** AST of the ST147 clone and its passage. **Table S2.** Pairwise SNP comparison between ST147 strains in this study. The numbers depict differences in SNPs exhibited by each strain pair. **Figure S1.** Growth curves of the ST147 strains. **Figure S2.** Serum killing assays of the ST147 isolates. **Figure S3.** Virulence of the MDR-ST147-hvKp evaluated by the Galleria mellonella model. **Figure S4.** Phylogenetic analysis of ST147 Kp in our hospital and the published ST147 genomes from GenBank database. The red dots adjacent to the tips of the tree represent MDR strains, the outer two rings of color bars represent resistance score and virulence score calculated by kleborate software, and the outer blue dots represent hvKp strains based on the presence of the five virulence associated genes including *peg-344, iroB, iucA, rmpA, *and *rmpA2*. **Figure S5.** Comparison between plasmid pPEKP4265-70 encoding *bla*OXA-48 found in this study and similar plasmids found in the online NCBI database. The outmost circle of arrows indicate the genes of reference plasmid pPEKP4265-70 used for comparison (red: AMR genes; green: integrase, recombinase, and transposase genes; purple: transfer associated genes; orange: gray: other functions). **Figure S6.** Comparison between plasmid pPEKP4265-152 encoding *bla*CTX-M-15 found in this study and similar plasmids found in the online NCBI database. The outmost circle of arrows indicate the genes of reference plasmid pPEKP4265-152 used for comparison (red: AMR genes; green: integrase, recombinase, and transposase genes; purple: transfer associated genes; orange: gray: other functions).

## Data Availability

The datasets used and/or analysed during the current study are available from the corresponding author on reasonable request.

## References

[1] Wang M, Earley M, Chen L, Hanson BM, Yu Y, Liu Z (2021). Clinical outcomes and bacterial characteristics of carbapenem-resistant Klebsiella pneumoniae complex among patients from different global regions (CRACKLE-2): a prospective, multicentre, cohort study. Lancet Infect dis.

[2] Wyres KL, Holt KE (2018). *Klebsiella pneumoniae* as a key trafficker of drug resistance genes from environmental to clinically important bacteria. Curr Opin Microbiol.

[3] Russo TA, Marr CM (2019). Hypervirulent *Klebsiella pneumoniae*. Clin Microbiol Rev.

[4] Abdul-Hamid A, Bailey SJ (2013). *Klebsiella pneumoniae* liver abscess and endophthalmitis. BMJ Case Rep.

[5] Gu D, Dong N, Zheng Z, Lin D, Huang M, Wang L (2018). A fatal outbreak of ST11 carbapenem-resistant hypervirulent *Klebsiella pneumoniae* in a Chinese hospital: a molecular epidemiological study. Lancet Infect Dis.

[6] Liu C, Du P, Xiao N, Ji F, Russo TA, Guo J (2020). Hypervirulent *Klebsiella pneumoniae* is emerging as an increasingly prevalent *K. pneumoniae* pathotype responsible for nosocomial and healthcare-associated infections in Beijing, China. Virulence.

[7] Damjanova I, Toth A, Paszti J, Hajbel-Vekony G, Jakab M, Berta J (2008). Expansion and countrywide dissemination of ST11, ST15 and ST147 ciprofloxacin-resistant CTX-M-15-type beta-lactamase-producing *Klebsiella pneumoniae* epidemic clones in Hungary in 2005–the new ‘MRSAs’?. J Antimicrob Chemother.

[8] Peirano G, Lascols C, Hackel M, Hoban DJ, Pitout JD (2014). Molecular epidemiology of *Enterobacteriaceae* that produce VIMs and IMPs from the SMART surveillance program. Diagn Microbiol Infect Dis.

[9] Giakkoupi P, Papagiannitsis CC, Miriagou V, Pappa O, Polemis M, Tryfinopoulou K (2011). An update of the evolving epidemic of blaKPC-2-carrying *Klebsiella pneumoniae* in Greece (2009–10). J Antimicrob Chemother.

[10] Falcone M, Giordano C, Barnini S, Tiseo G, Leonildi A, Malacarne P (2020). Extremely drug-resistant NDM-9-producing ST147 *Klebsiella pneumoniae* causing infections in Italy, May 2020. Euro surveill.

[11] Martin MJ, Corey BW, Sannio F, Hall LR, MacDonald U, Jones BT (2021). Anatomy of an extensively drug-resistant *Klebsiella pneumoniae* outbreak in Tuscany, Italy. Proc Natl Acad Sci U S A.

[12] Di Pilato V, Henrici De Angelis L, Aiezza N, Baccani I, Niccolai C, Parisio EM (2022). Resistome and virulome accretion in an NDM-1-producing ST147 sublineage of *Klebsiella pneumoniae* associated with an outbreak in Tuscany, Italy: a genotypic and phenotypic characterisation. Lancet Microbe.

[13] Peirano G, Chen L, Kreiswirth BN, Pitout JDD (2020). Emerging antimicrobial-resistant high-risk *Klebsiella pneumoniae* clones ST307 and ST147. Antimicrob Agents Chemother.

[14] Zhang Y, Jin L, Ouyang P, Wang Q, Wang R, Wang J (2019). Evolution of hypervirulence in carbapenem-resistant *Klebsiella pneumoniae* in China: a multicentre, molecular epidemiological analysis. J Antimicrob Chemothe.

[15] Falcone M, Tiseo G, Arcari G, Leonildi A, Giordano C, Tempini S (2022). Spread of hypervirulent multidrug-resistant ST147 *Klebsiella pneumoniae* in patients with severe COVID-19: an observational study from Italy, 2020–21. J Antimicrob Chemother.

[16] Liu C, Shi J, Guo J (2018). High prevalence of hypervirulent *Klebsiella pneumoniae* infection in the genetic background of elderly patients in two teaching hospitals in China. Infect Drug Resist.

[17] Russo TA, Olson R, Fang CT, Stoesser N, Miller M, MacDonald U (2018). Identification of biomarkers for differentiation of hypervirulent *Klebsiella pneumoniae* from classical *K. pneumoniae*. J clin microbiol.

[18] Liu C, Guo J (2018). Characteristics of ventilator-associated pneumonia due to hypervirulent *Klebsiella pneumoniae* genotype in genetic background for the elderly in two tertiary hospitals in China. Antimicrob Resist Infect Control.

[19] Liu C, Du P, Zhao J, Li B, Wang C, Sun L (2020). Phenotypic and genomic characterization of virulence heterogeneity in multidrug-resistant ST11 *Klebsiella pneumoniae* during inter-host transmission and evolution. Infect Drug Resist.

[20] Jousset AB, Bonnin RA, Rosinski-Chupin I, Girlich D, Cuzon G, Cabanel N (2018). A 4.5-year within-patient evolution of a colistin-resistant *Klebsiella pneumoniae* carbapenemase-producing *K. pneumoniae* sequence type 258. Clin Infect Dis Off Publicat Infect Dis Soc Am.

[21] Liu C, Pan F, Guo J, Yan W, Jin Y, Liu C (2016). Hospital acquired pneumonia due to *Achromobacter* spp. in a geriatric ward in China: clinical characteristic, genome variability, biofilm production, antibiotic resistance and integron in isolated strains. Front microbiol.

[22] Zhang Y, Wang X, Wang Q, Chen H, Li H, Wang S (2021). Emergence of tigecycline nonsusceptible and IMP-4 carbapenemase-producing K2-ST65 hypervirulent *Klebsiella pneumoniae* in China. Microbiol spectr.

[23] Hirabayashi A, Dao TD, Takemura T, Hasebe F, Trang LT, Thanh NH (2021). A transferable IncC-IncX3 hybrid plasmid cocarrying blaNDM-4, tet(X), and tmexCD3-toprJ3 confers resistance to carbapenem and tigecycline. mSphere.

[24] Bankevich A, Nurk S, Antipov D, Gurevich AA, Dvorkin M, Kulikov AS (2012). SPAdes: a new genome assembly algorithm and its applications to single-cell sequencing. J Comput Biol J Comput Mol Cell Biol.

[25] Bortolaia V, Kaas RS, Ruppe E, Roberts MC, Schwarz S, Cattoir V (2020). ResFinder 4.0 for predictions of phenotypes from genotypes. T J antimicrob chemother.

[26] Chen L, Yang J, Yu J, Yao Z, Sun L, Shen Y (2005). VFDB: a reference database for bacterial virulence factors. Nucleic Acids Res.

[27] Siguier P, Perochon J, Lestrade L, Mahillon J, Chandler M (2006). ISfinder: the reference centre for bacterial insertion sequences. Nucleic Acids Res.

[28] Carattoli A, Hasman H (2020). PlasmidFinder and in silico pMLST: identification and typing of plasmid replicons in whole-genome sequencing (WGS). Methods Mol Biol.

[29] Wick RR, Judd LM, Gorrie CL, Holt KE (2017). Unicycler: resolving bacterial genome assemblies from short and long sequencing reads. PLoS Comput Biol.

[30] Lam MMC, Wick RR, Watts SC, Cerdeira LT, Wyres KL, Holt KE (2021). A genomic surveillance framework and genotyping tool for *Klebsiella pneumoniae* and its related species complex. Nat Commun.

[31] Langmead B, Salzberg SL (2012). Fast gapped-read alignment with Bowtie 2. Nat Methods.

[32] Croucher NJ, Page AJ, Connor TR, Delaney AJ, Keane JA, Bentley SD (2015). Rapid phylogenetic analysis of large samples of recombinant bacterial whole genome sequences using Gubbins. Nucleic Acids Res.

[33] Price MN, Dehal PS, Arkin AP (2010). FastTree 2–approximately maximum-likelihood trees for large alignments. PLoS ONE.

[34] Biedrzycka M, Urbanowicz P, Guzek A, Brisse S, Gniadkowski M, Izdebski R (2021). Dissemination of *Klebsiella pneumoniae* ST147 NDM-1 in Poland, 2015–19. J Antimicrob Chemother.

[35] Lapp Z, Crawford R, Miles-Jay A, Pirani A, Trick WE, Weinstein RA (2021). Regional spread of blaNDM-1-containing *Klebsiella pneumoniae* ST147 in post-acute care facilities. Clin Infect Dis Off Publicat Infect Dis Soc Am.

[36] Wang X, Xu X, Li Z, Chen H, Wang Q, Yang P (2014). An outbreak of a nosocomial NDM-1-producing *Klebsiella pneumoniae* ST147 at a teaching hospital in mainland China. Microb Drug Resist.

[37] Zou H, Shen Y, Li C, Li Q (2022). Two Phenotypes of *Klebsiella pneumoniae* ST147 outbreak from neonatal sepsis with a slight increase in virulence. Infect Drug Resist.

[38] Wang Q, Wang X, Wang J, Ouyang P, Jin C, Wang R (2018). Phenotypic and genotypic characterization of carbapenem-resistant *Enterobacteriaceae*: data from a longitudinal large-scale CRE study in China (2012–2016). Clin Infect Dis Off Publicat Infect Dis Soc Am.

[39] Wyres KL, Wick RR, Judd LM, Froumine R, Tokolyi A, Gorrie CL (2019). Distinct evolutionary dynamics of horizontal gene transfer in drug resistant and virulent clones of *Klebsiella pneumoniae*. PLoS Genet.

[40] Rodrigues C, Desai S, Passet V, Gajjar D, Brisse S (2022). Genomic evolution of the globally disseminated multidrug-resistant *Klebsiella pneumoniae* clonal group 147. Microb genom.

[41] Xie M, Yang X, Xu Q, Ye L, Chen K, Zheng Z (2021). Clinical evolution of ST11 carbapenem resistant and hypervirulent *Klebsiella pneumoniae*. Commun biol.

[42] Yang X, Wai-Chi Chan E, Zhang R, Chen S (2019). A conjugative plasmid that augments virulence in *Klebsiella pneumoniae*. Nat microbiol.

[43] Dong N, Lin D, Zhang R, Chan EW, Chen S (2018). Carriage of blaKPC-2 by a virulence plasmid in hypervirulent *Klebsiella pneumoniae*. J Antimicrob Chemother.

[44] Du P, Liu C, Fan S, Baker S, Guo J (2022). The role of plasmid and resistance gene acquisition in the emergence of ST23 multi-drug resistant hypervirulent *Klebsiella pneumoniae*. Microbiol spectr.

[45] Zautner AE, Bunk B, Pfeifer Y, Sproer C, Reichard U, Eiffert H (2017). Monitoring microevolution of OXA-48-producing *Klebsiella pneumoniae* ST147 in a hospital setting by SMRT sequencing. J Antimicrob Chemother.

[46] Huang W, Wang G, Sebra R, Zhuge J, Yin C, Aguero-Rosenfeld ME (2017). Emergence and evolution of multidrug-resistant *Klebsiella pneumoniae* with both blaKPC and blaCTX-M integrated in the chromosome. Antimicrob Agents Chemother.

