# Supplementary material for: Fusion plasmid enhanced the endemic extensively drug resistant Klebsiella pneumoniae clone ST147 harbored blaOXA-48 to acquire the hypervirulence and cause fatal infection
Source: Ann Clin Microbiol Antimicrob. 2023 Feb 14;22:11. doi: 10.1186/s12941-022-00551-1 (PMC9927049; doi:10.1186/s12941-022-00551-1)

Additional file 1: Table S1. AST of the ST147 clone and its passage.

Items	P4	**PEKP 4035**	PEK P 4225	PEKP 4243	**PEKP 4265**	PEK P507 8	**10th Passage -4035**	10th Passage -4265
TZP	32	**≥128**	≤4	≤4	**≥128**	≥ 128	**≥128**	≤4
CAZ	32	**≥64**	32	4	**≥64**	32	**≥64**	8
CSL	16	**≥64**	≤8	16	**≥64**	≥64	**≥64**	≤8
FEP	≥32	**≥32**	≥32	2	**≥32**	≥32	**≥32**	2
ATM	≥64	**≥64**	16	2	**4**	≥64	**≥64**	4
IPM	≤ 0.25	**≥16**	≤ 0.25	≤0.25	**≥16**	≤ 0.25	**8**	≤0.25
MEM	≤ 0.25	**≥16**	≤ 0.25	≤0.25	10*	≤ 0.25	**≥16**	≤0.25
AMK	≤2	**≥64**	≤2	≤2	**≥64**	≤2	**≥64**	≤2
TOB	8	**≥16**	≤1	8	≤1	8	**≥16**	≤1
CIP	≥4	≥4	≥4	≥4	≥4	≥4	**≥4**	≥4
LVX	≥8	≥8	≥8	≥8	4	≥8	**≥8**	≥8
MNO	≥16	**≥16**	≤1	8	18*	8	**≥16**	≤1
TGC	≥8	**4**	≤0.5	2	1	2	**4**	≤0.5
POL	≤0.5	**≤0.5**	≤0.5	≤0.5	≤0.5	≤0.5	**≤0.5**	≤0.5
SXT	≥320	**≤20**	≥	≥320	≥320	≥	**≤20**	≥320

			320			320		
CZA	26	**25**	29	28	28	28	**21**	28
FDC	23	**21**	25	25	23	25	**21**	23

*： AST was determined by the KB method.

TZP: piperacillin/tazobactam; CAZ: ceftazidime; CSL: cefperazone-sulbactam; FEP: cefepime; ATM: aztreonam; IPM: imipenem; MEM: meropenem; AMK: amikacin; TOB: tobramycin; CIP: ciprofloxacin; LVX: levofloxacin; MNO: minocycline; TGC: tigecycline; POL: polymyxin; SXT: trimethoprim/sulfamethoxazole; CZA: ceftazidime/avibactam; FDC: cefiderocol.

Additional file 1: Table S2. Pairwise SNP comparison between ST147 strains in this study. The numbers depict differences in SNPs exhibited by each strain pair.

Strain ID	P4	PEKP4243	PEKP4035	PEKP4265	PEKP5078	PEKP4225
P4	0	139	151	157	657	1414
PEKP4243	139	0	136	148	674	1411
PEKP4035	151	136	0	20	674	1425
PEKP4265	157	148	20	0	676	1427
PEKP5078	657	674	674	676	0	1551
PEKP4225	1414	1411	1425	1427	1551	0

Additional file 1: Figure S1. Growth curves of the ST147 strains.

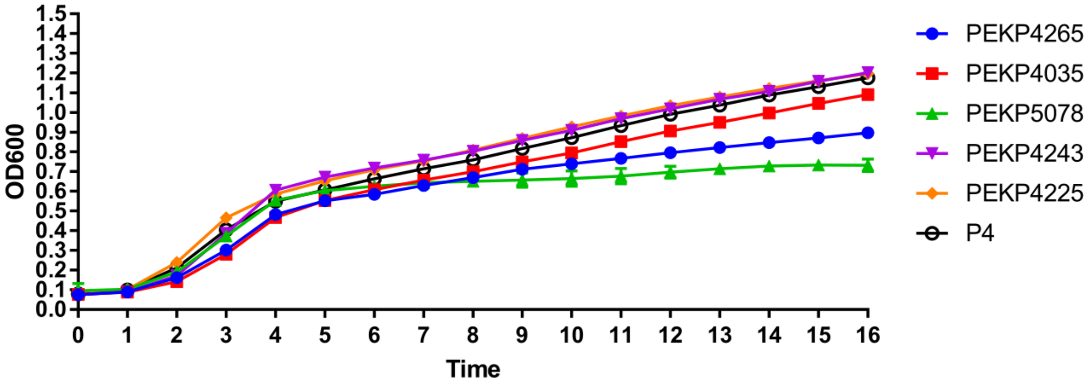

Additional file 1: Figure S2. Serum killing assays of the ST147 isolates.

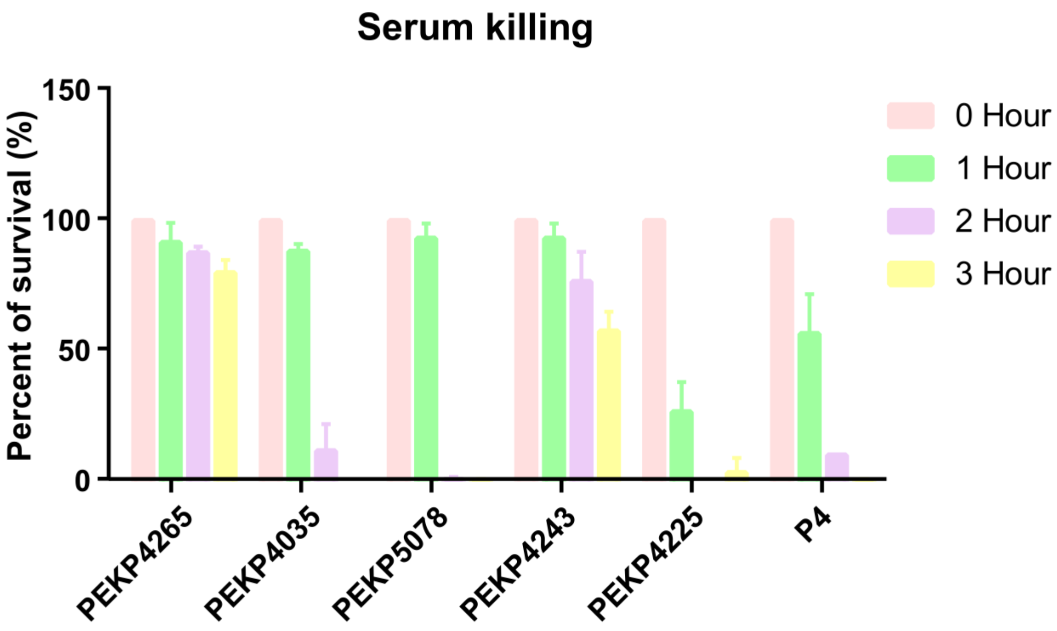

Additional file 1: Figure S3. Virulence of the MDR-ST147-hvKp evaluated by the Galleria mellonella model.

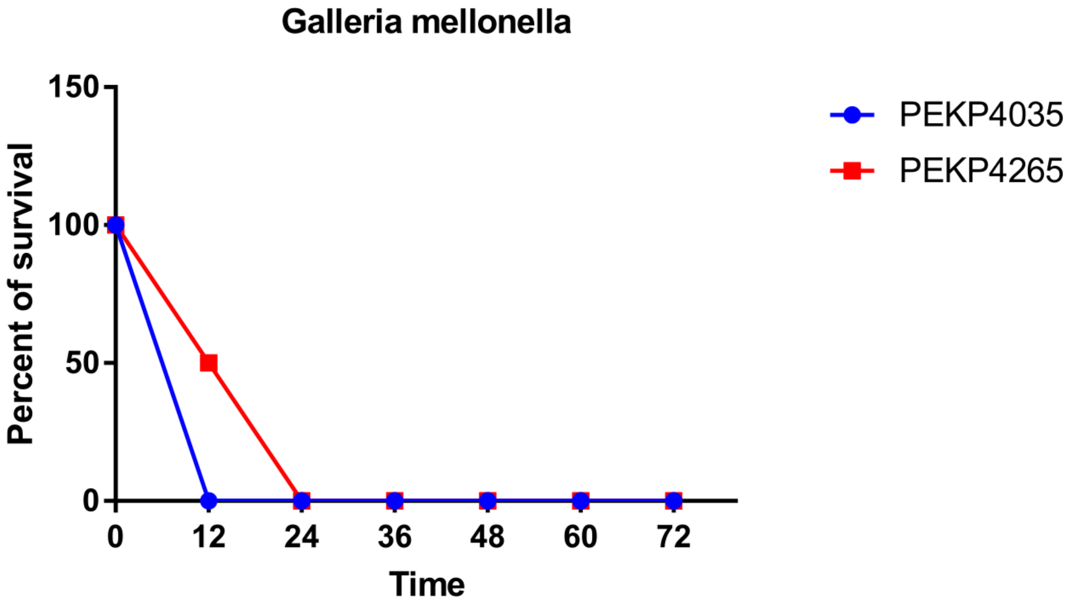

Additional file 1: Figure S4. Phylogenetic analysis of ST147 Kp in our hospital and the published ST147 genomes from GenBank database. The red dots adjacent to the tips of the tree represent MDR strains, the outer two rings of color bars represent resistance score and virulence score calculated by kleborate software, and the outer blue dots represent hvKp strains based on the presence of the five virulence associated genes including *peg-344, iroB, iucA, rmpA,* and *rmpA2*.

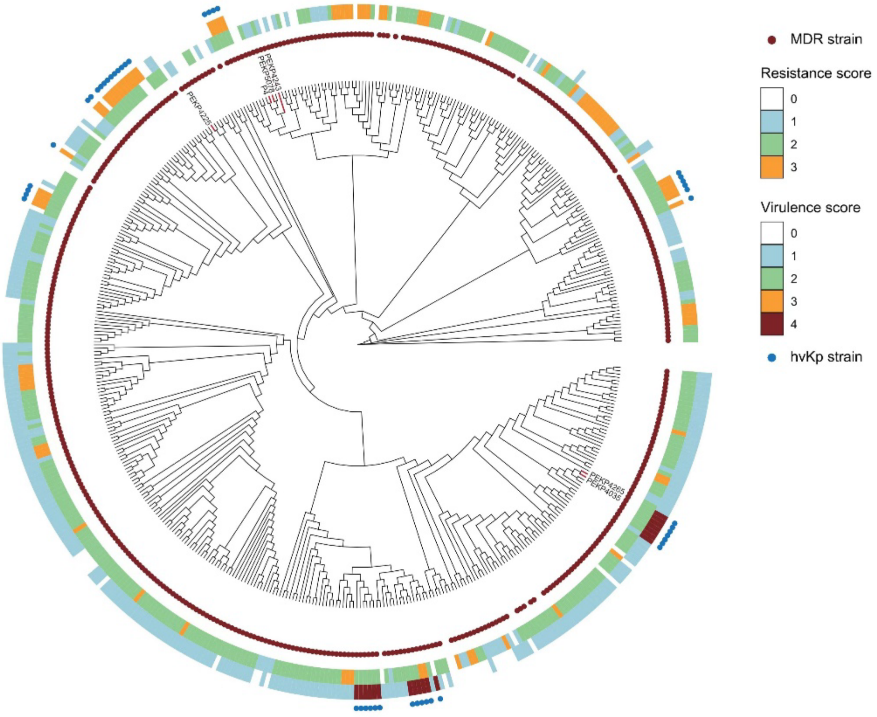

Additional file 1: Figure S5. Comparison between plasmid pPEKP4265-70 encoding *bla*$_{OXA-48}$ found in this study and similar plasmids found in the online NCBI database. The outmost circle of arrows indicate the genes of reference plasmid pPEKP4265-70 used for comparison (red: AMR genes; green: integrase, recombinase, and transposase genes; purple: transfer associated genes; orange: gray: other functions).

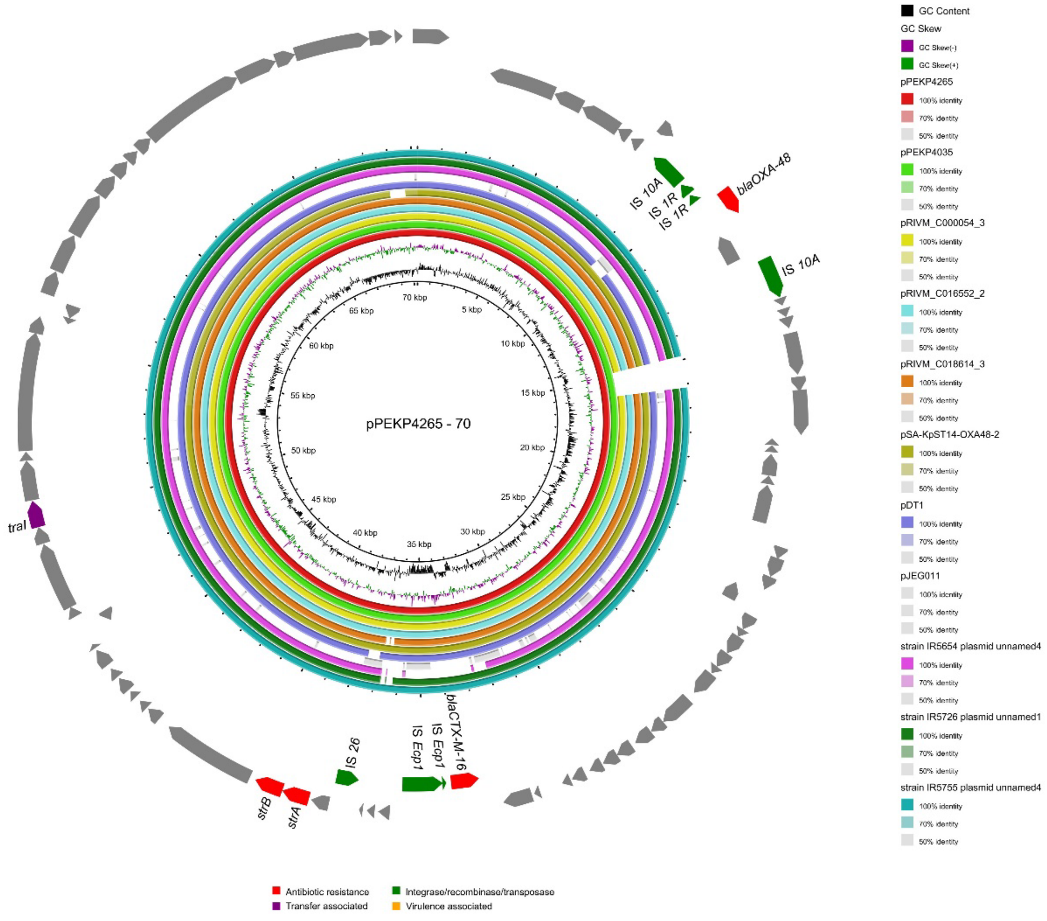

Additional file 1: Figure S6**.** Comparison between plasmid pPEKP4265-152 encoding *bla*$_{CTX-M-15}$ found in this study and similar plasmids found in the online NCBI database. The outmost circle of arrows indicate the genes of reference plasmid pPEKP4265-152 used for comparison (red: AMR genes; green: integrase, recombinase, and transposase genes; purple: transfer associated genes; orange: gray: other functions).

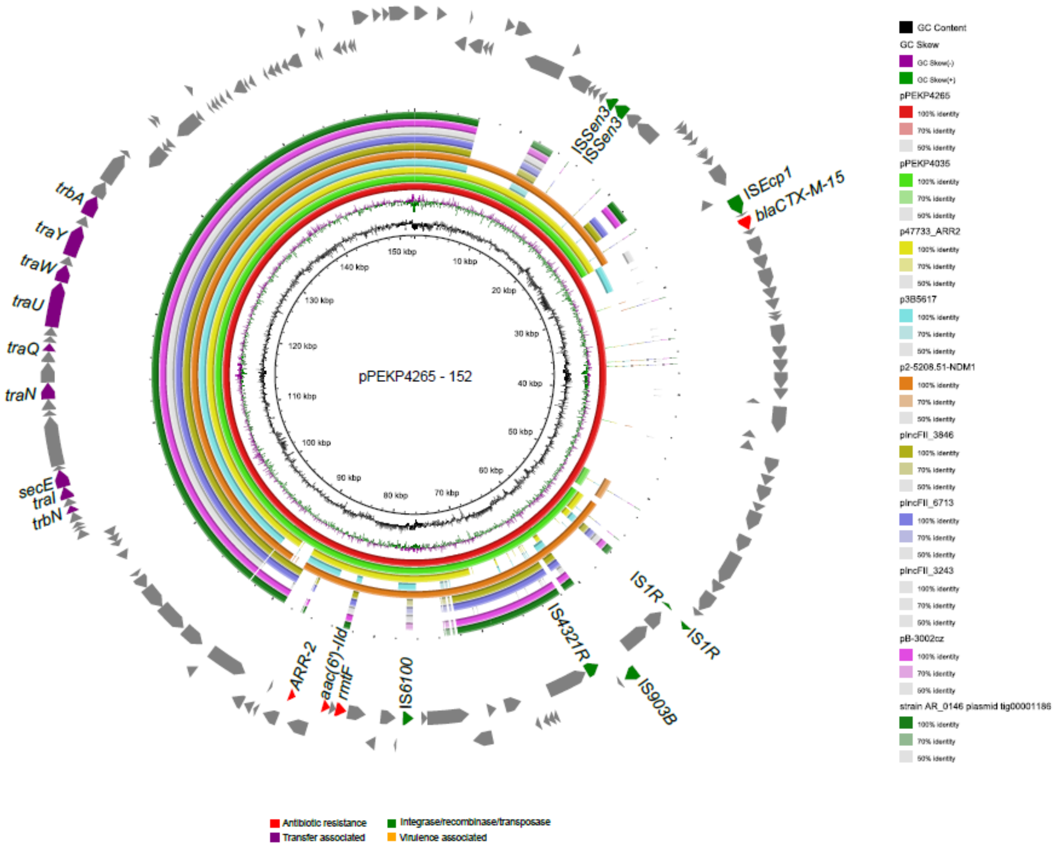

Supplement: Supplementary file 1 — Additional file 1: Table S1. AST of the ST147 clone and its passage. Table S2. Pairwise SNP comparison between ST147 strains in this study. The numbers depict differences in SNPs exhibited by each strain pair. Figure S1. Growth curves of the ST147 strains. Figure S2. Serum killing assays of the ST147 isolates. Figure S3. Virulence of the MDR-ST147-hvKp evaluated by the Galleria mellonella model. Figure S4. Phylogenetic analysis of ST147 Kp in our hospital and the published ST147 genomes from GenBank database. The red dots adjacent to the tips of the tree represent MDR strains, the outer two rings of color bars represent resistance score and virulence score calculated by kleborate software, and the outer blue dots represent hvKp strains based on the presence of the five virulence associated genes including peg-344, iroB, iucA, rmpA, and rmpA2. Figure S5. Comparison between plasmid pPEKP4265-70 encoding blaOXA-48 found in this study and similar plasmids found in the online NCBI database. The outmost circle of arrows indicate the genes of reference plasmid pPEKP4265-70 used for comparison (red: AMR genes; green: integrase, recombinase, and transposase genes; purple: transfer associated genes; orange: gray: other functions). Figure S6. Comparison between plasmid pPEKP4265-152 encoding blaCTX-M-15 found in this study and similar plasmids found in the online NCBI database. The outmost circle of arrows indicate the genes of reference plasmid pPEKP4265-152 used for comparison (red: AMR genes; green: integrase, recombinase, and transposase genes; purple: transfer associated genes; orange: gray: other functions). [file 12941_2022_551_MOESM1_ESM.pdf]
